# Clinical phenotypes of *MAGEL2* mutations and deletions

**DOI:** 10.1186/1750-1172-9-40

**Published:** 2014-03-25

**Authors:** Karin Buiting, Nataliya Di Donato, Jasmin Beygo, Susanne Bens, Maja von der Hagen, Karl Hackmann, Bernhard Horsthemke

**Affiliations:** 1 Institut für Humangenetik, Universitätsklinikum Essen, Universität Duisburg-Essen, Essen, Germany; 2 Institut für Klinische Genetik, Medizinische Fakultät Carl Gustav Carus, TU Dresden, Dresden, Germany; 3 Institute of Human Genetics, University Hospital Schleswig-Holstein, Campus Kiel & Christian-Albrechts-University Kiel, Kiel, Germany; 4 Department of Neuropediatrics, Technical University Dresden, Dresden, Germany

## Letter to the Editor

Although it has long been known that Prader-Willi syndrome (PWS) is caused by the loss of function of imprinted, paternally expressed genes in 15q11q13, the contribution of the different genes within this region has not yet been completely resolved. Based on the identification of rare deletions affecting only the snoRNA gene cluster *SNORD116* it has been suggested that this is the major locus [1-3]. Recently, Schaaf et al. have described truncating mutations of *MAGEL2* in four patients with a broad range of clinical phenotypes [4]. The authors conclude that "*MAGEL2* loss of function can contribute to several aspects of the PWS phenotype". While this may be true, we think that the available data are not sufficient to justify this conclusion.

**Figure 1:**
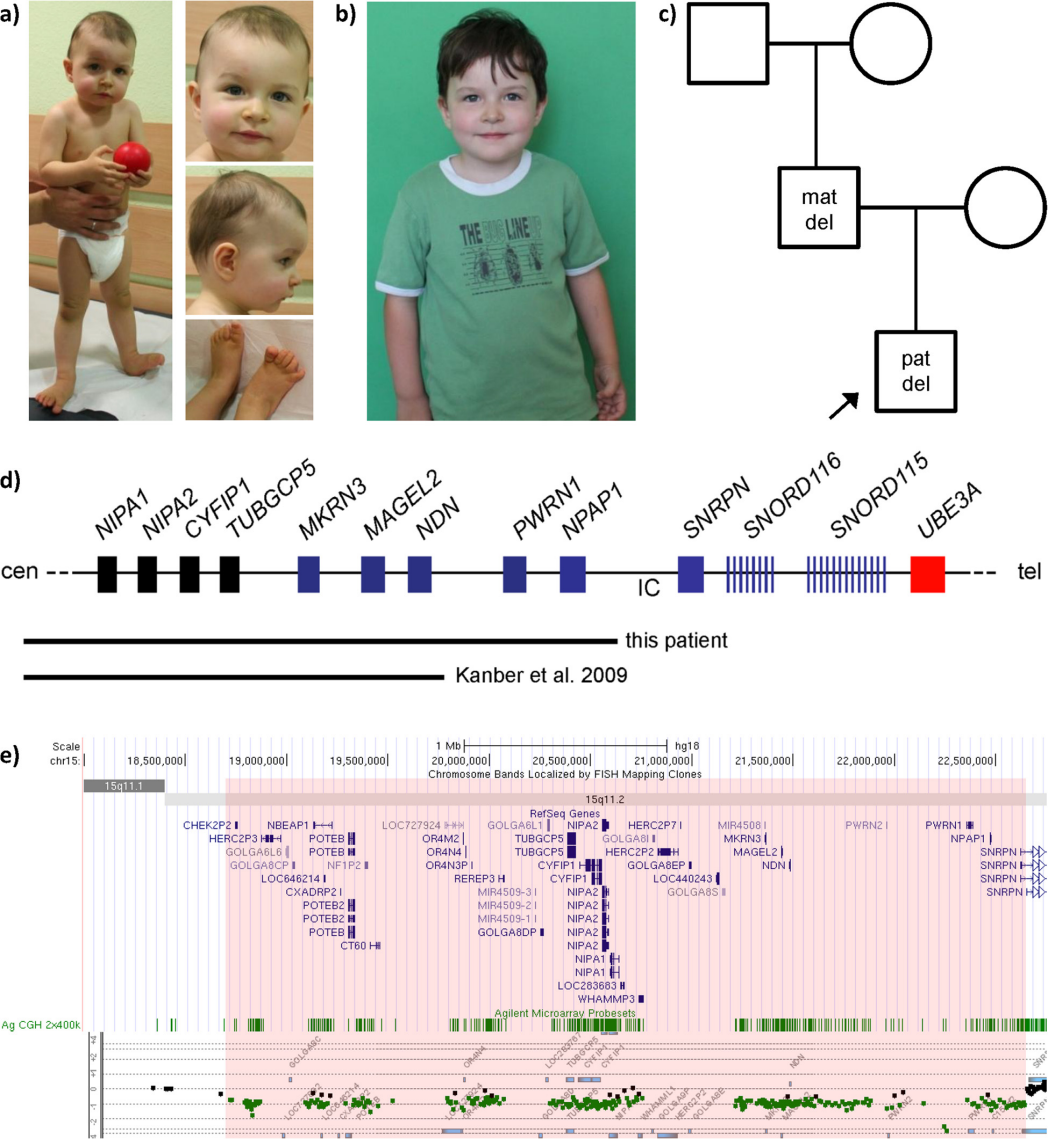
**Clinical and molecular findings in the patient. a)** The patient at the age of five months and **b)** at 3 3/12 years. **c)** Pedigree of the family. The patient has the deletion on his paternal chromosome, whereas his father has the deletion on his maternal chromosome. **d)** Schematic overview of the chromosomal region 15q12. Blue boxes and bars, paternally expressed genes; red box, maternally expressed gene; black boxes, biparentally expressed genes; IC, imprinting center. The deletion is indicated by a black horizontal bar. Not drawn to scale. **e)** CGH Array results of the patient.The chromosomal localisation together with the RefSeq genes are shown in the upper part of the plot. The location of the Agilent microarray probesets is given below (green). The region highlighted in light red is deleted. The bottom part shows the gene dosage detected by the probesets (squares). Green - reduced dosage, black - normal dosage, red – increased dosage. Genomic coordinates are according to hg18. Please note that in the UCSC browser the genes *NIPA1* to *TUBGCP5* are in the wrong order due to flanking sequence gaps.

We have recently seen a 3-year-old boy with a paternally inherited deletion of ~ 3.9 Mb that includes *MAGEL2*, but not the *SNRPN/SNORD116* locus (Figure 1 and Additional file 1: Figure S1). Apart from delayed motor skills, the boy is asymptomatic (for a detailed clinical description see Additional file 1). This is the second individual with a *MAGEL2* deletion who certainly does not have PWS; the first one was also described by our group [5]. Here we offer an explanation for the apparently discrepant findings, which is also important for deciphering the role of candidate genes in PWS and other contiguous gene syndromes.

Usually, exome sequencing is performed to identify a gene that is affected in several patients with the same disease. The identification of such a gene is a strong indication that a mutation in this gene causes the disease. Schaaf et al. have started their study with a patient of unknown clinical diagnosis, whose genome was investigated under a *de novo* model only. The other three patients were identified by searching a clinical exome data base. Apparently, an exome-wide analysis under different genetic models was not performed in these patients. Therefore, the number of potentially pathogenic variants in these patients is unknown. In this situation, it is difficult to prove causality, especially when there is no consistent phenotype (since each of the Holm's criteria for PWS refers to a rather common and unspecific clinical sign, many patients with diverse disorders fulfill some of them; these should not be called "PWS phenotypes"). The paternal origin of the *MAGEL2* mutations does not prove causality, because the majority of point mutations occur during spermatogenesis. In summary, it is possible that the *MAGEL2* mutations are innocent bystanders and that the patients have autosomal recessive or X-linked recessive disease (note that all patients are male).

Even if the *MAGEL2* mutations were causally related to the clinical phenotypes of the patients described by Schaaf et al., it is still possible that they do not contribute to PWS, and there is a precedent for this. In fact, *MAGEL2* is not the first protein-coding gene in the PWS region found to be mutated. The first one is *MKRN3* (Figure 1d), which was found to be mutated in patients with central precocious puberty [6]. In contrast to these patients, patients with PWS typically have incomplete or delayed puberty. The finding that *MKRN3* loss of function alone causes central precocious puberty, but not in combination with the loss of function of the *SNORD116* genes, indicates that the *SNORD116* loss of function is epistatic to *MKRN3* loss of function, probably because the *SNORD116* genes act developmentally upstream of *MKRN3*.

Another possibility is that there is leaky expression of the maternal *MAGEL2* allele in a subset of neurons in patients with a paternal *MAGEL2* deletion (our patient and PWS patients), but not in patients with a truncating *MAGEL2* mutation (the patients described by Schaaf et al.). A precedent for this situation is the recent finding of stochastic loss of silencing of the imprinted *Ndn/NDN* allele [7]. These authors find weak expression of the maternal *Ndn* allele in mice with a targeted deletion of the *Ndn* gene that includes the promoter but not in mice with a targeted deletion that does not include the promoter, probably because of promoter competition. We note that the four patients described by Schaaf et al. have an intact promoter on the paternal allele, whereas our two patients and the majority of PWS patients don't.

We conclude that it is important to distinguish between point mutations and whole gene deletions and that the effect of the genes in the PWS chromosomal region may be epistatic rather than additive. Therefore, the role of *MAGEL2* in PWS remains unclear.

## Consent

Written informed consent was obtained from the parents of the patient for publication of this manuscript and any accompanying images. A copy of the written consent is available for review by the Editor-in-Chief of this journal.

## Competing interests

The authors declare no competing interests.

## Authors’ contributions

KB and BH supervised this project. KB and JB planned the experiments and analyzed data. MvdH carried out the clinical evaluation and the clinical diagnostic workup. ND performed dysmorphological evaluation, genetic counseling, collected samples and initiated genetic testing. KH performed and analyzed CGH array analysis. SB performed and analyzed custom array analysis. BH wrote the manuscript. All authors reviewed and approved the final version of the manuscript.

## Supplementary Material

Additional file 1 Supplementary information. Click here for file
