# Supplementary material for: Clinical phenotypes of MAGEL2 mutations and deletions
Source: Orphanet J Rare Dis. 2014 Mar 25;9:40. doi: 10.1186/1750-1172-9-40 (PMC3987887; doi:10.1186/1750-1172-9-40)
Supplement: Additional file 1 — Supplementary information. [file 1750-1172-9-40-S1.pdf]

Supplementary information

Clinical description

The patient was born after an uneventful pregnancy in the 40+2 week of gestation to healthy non-consanguineous parents. His birth measurements were all within the normal range with length 48 cm (-1.96 SD), weight 3010g (-1.39 SD) and head circumference 34.5 cm (-0.85 SD). He presented with a transient muscular hypotonia associated with mild feeding difficulties in infancy (no tube feeding required) and thoracal kyphosis. Minor elevation of the creatinkinase was observed. Motor milestones were just within the normal range (e.g. unsupported walking with 18 months), but fine and gross motor skills were slightly delayed. Intellectual development was normal (first words with 14 months). At the age of 3 years and 3 months his measurements were: length 102.5 cm (1.45 SD), weight 17.3 kg (50-75 percentile) and head circumference 50.3 cm (-0.37 SD) all within the normal range. Due to the deletion of *MKRN3*, the patient may develop precocious puberty, as did the patient described by Kanber et al.[2].

Material and Methods

Blood samples were obtained after written informed consent. For the patient we obtained written informed consent from his parents. The patient’s parents provided written informed consent for publication of case details. The study was approved by the ethics committees of the University Hospital Essen.

Array CGH

Cytogenetic analyzes revealed a normal male karyotype 46,XY. Genome-wide investigation of copy number alterations was performed on DNA from peripheral blood by array comparative genomic hybridization (CGH) using the Agilent Human Genome CGH Microarray Kit 2x400k (design 21850) according to the manufacturer’s protocol (Agilent Technologies, Santa Clara, USA). 1µg of the patient’s DNA was used as test DNA and male MegaPool Reference DNA was used as reference DNA (Kreatech Diagnostics, NL). Data analysis was conducted using Agilent’s Genomic Workbench Standard Edition Version 5.0.14. The ADM-2 algorithm was applied with a threshold of 5.9. A minimum of 4 consecutive probes had to be affected to make a call. Scanning was carried out on an

Agilent microarray scanner and raw data were processed by Agilent's Feature Extraction Software Version 9.5.

MS-MLPA

Verification of the deletion was conducted using (MS)-MLPA (methylation specific-multiplex ligation probe amplification; MRC Holland, Netherlands; Kit ME028-B2) according to the manufacturer's manual. Data analysis was performed using GeneMarker software (Softgenetics, USA).

Custom Array

For high-resolution characterization of the breakpoints a customized array CGH was used. The array was designed using the eArray software (Agilent Technologies, Inc, Santa Clara, CA, USA). In total 33,314 High definition (HD) and Tiling probes (probe length 60 nt) were selected for a 4x44k format covering, among other imprinted loci, the *SNRPN* containing region on chromosome 15 (chr15:23,810,000 – 25,296,000, hg19).

A backbone of 7,021 randomly selected oligonucleotides covering the whole genome in addition to 4,885 technical control probes was applied. Experimental procedures were performed according to the manufacturer's instructions. 1 µg DNA was used as template and hybridized against a normal female DNA template as reference control. Arrays were scanned with the DNA Microarray Scanner (Agilent Technologies) at a resolution of 5 µm double pass. Raw data was extracted and analyzed with the AGW Feature Extraction 10.10.1.1. and the Agilent Genomic Workbench Standard Edition 6.5.0.58 software packages.

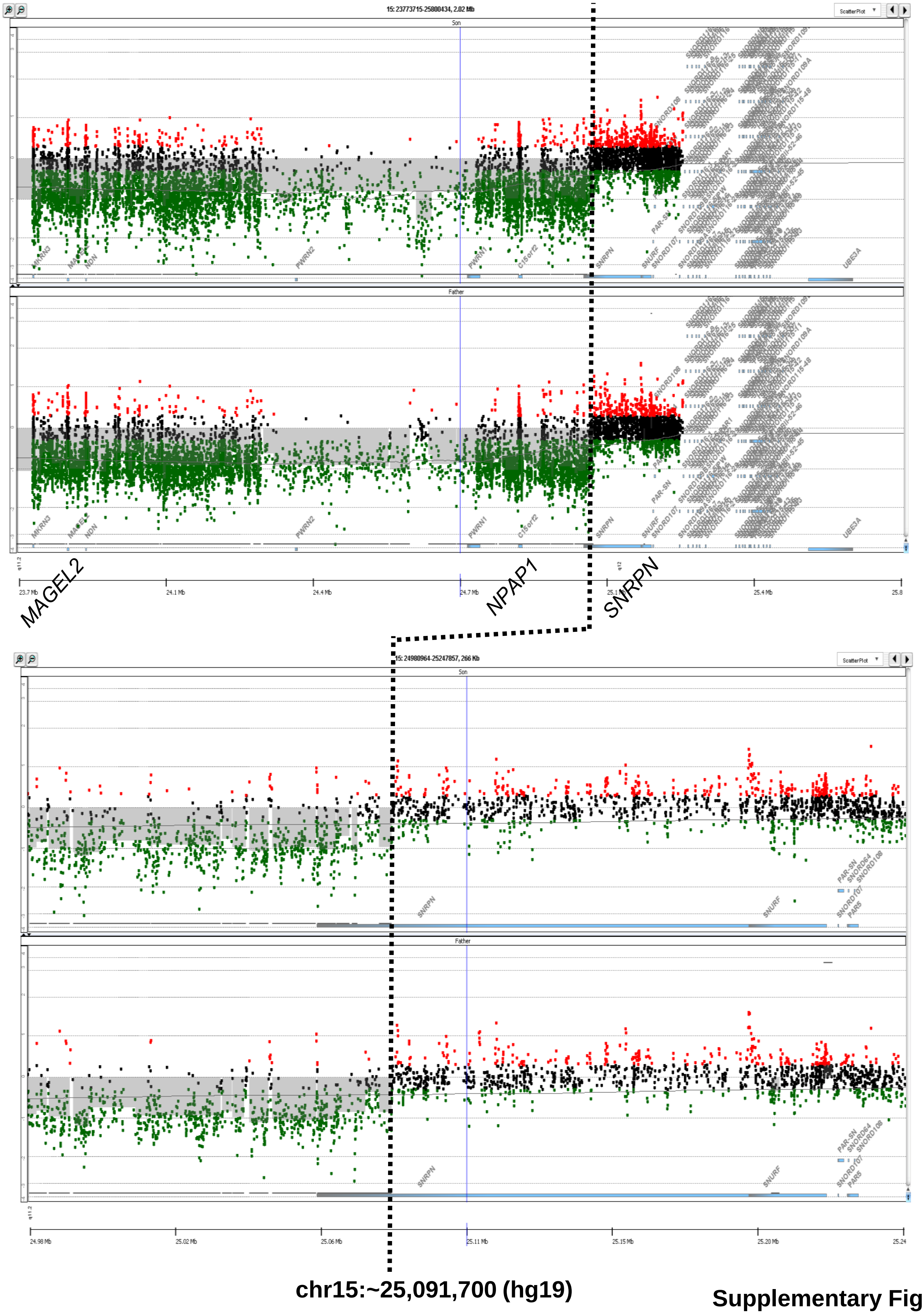

Supplementary Figure 1

Supplementary Figure 1. Results of the customized array CGH of the patient (Son) and his father (Father). The upper part of the figure provides an overview of chromosomal region 15q11.2q12 in a 2.02 Mb window. Black circles represent log2 ratios in a balanced range, green circles indicate log2 ratios below -0.3 and red circles correspond to log2 ratios above 0.3. Deleted regions detected by the software are shaded in grey. Dashed black line indicates the telomeric border of the deletion [assigned to approximately chr15:~25,091,700 (hg19), corresponding to chr15:~22,642,800 (hg18)]. The lower panel shows a blow-up of the distal breakpoint region in a 266 kb window.

Supplementary Table 1

Length (nt)	MLPA probes	P	F	GM
427	Reference 01p21	0.846	1.040	0.858
264	Reference 07q21	0.997	0.980	0.977
136	Reference 10q26	0.966	0.916	0.965
454	Reference 11p15	0.874	1.009	0.880
130	Reference 11q13	1.101	0.968	1.000
166	Reference 11q24	1.068	0.903	1.022
319	Reference 11q24	0.999	0.964	1.041
208	Reference 12q13	0.937	0.981	0.972
337	Reference 13q24	0.956	1.122	1.044
148	Reference 17q12	0.999	1.000	1.000
480	Reference 17q23	1.057	0.975	0.918
309	Reference 22q12	0.985	1.068	1.113
244	Reference 05p15	1.022	1.000	1.000
232	Reference 17q21	1.002	1.098	1.008
463	3p22	1.022	1.179	0.939
**434**	***NIPA1* BP1-BP2 region**	**0.517**	**0.566**	**0.898**
**154**	***TUBGCP5* BP1-BP2 region**	**0.527**	**0.474**	**0.998**
**172**	***MKRN3* exon 1**	**0.584**	**0.501**	**0.989**
**409**	***MAGEL2* exon 1**	**0.491**	**0.498**	**0.881**
**445**	***NDN* exon 1**	**0.515**	**0.535**	**0.899**
**419**	***NDN* exon 1**	**0.474**	**0.581**	**1.004**
**287**	***SNRPN* Exon u1B**	**0.524**	**0.517**	**1.034**
**238**	***SNRPN* Exon u1B***	**0.520**	**0.518**	**1.017**
278	*SNRPN* Intron u2	1.026	0.968	0.917
270	*SNRPN* Intron u2	0.982	1.031	1.048
256	*SNRPN* Exon u5 (AS-SRO)	1.089	0.987	1.000
391	*SNRPN* Exon u5 (AS-SRO)	1.007	1.161	1.020
250	*SNRPN* CpG island	1.021	0.835	1.000
178	*SNRPN* CpG island	0.984	0.950	1.017
190	*SNRPN* CpG island	0.996	0.885	1.000
142	*SNRPN* CpG island	1.113	0.955	1.000
294	*SNURF-SNRPN* exon 3	0.928	0.949	0.932
400	*SNURF-SNRPN* exon 7	1.050	1.052	0.954
214	*SNORD116* snoRNA cluster	0.820	0.970	0.977
472	*SNORD116* snoRNA cluster	0.820	1.030	0.862
328	*SNORD116* snoRNA cluster	0.984	1.057	0.960
355	*UBE3A* exon 13	0.978	1.027	0.986
301	*UBE3A* exon 8	0.964	1.090	1.017
160	*UBE3A* exon 7	1.069	0.925	1.007
197	*UBE3A* exon 6	0.997	0.929	1.000
373	*UBE3A* exon 5	1.006	1.008	1.025
184	*UBE3A* exon 1	1.055	0.899	0.967
366	*ATP10A* exon 5	1.076	1.088	0.975
226	*ATP10A* exon 1	0.958	1.049	0.960
220	*GABRB3* exon 12	0.959	0.939	1.075
382	*GABRB3* exon 10	0.829	0.984	0.852
202	*APBA2* Exon 14	0.941	1.069	1.138
346	*BLM* 15q26	0.958	1.112	1.012

Length (nt)	MS-MLPA probes	P	F
**419**	***NDN* exon 1**	**0.90**	**0.00**
178	*SNRPN* CpG island	0.59	0.58
190	*SNRPN* CpG island	0.57	0.60
142	*SNRPN* CpG island	0.51	0.51

Supplementary Table 1. Results of the MS-MLPA analysis. Gene dosage analysis confirmed the results obtained by CGH array analysis and revealed that the patient and the father have a 50% reduced dosage for the most centromeric loci of chromosome 15, whereas all other probes for chromosome 15 and all reference probes from other chromosomes showed a normal dosage. Methylation analysis showed a normal methylation level of approximately 50% in both patient and father for three methylation sensitive *SNRPN* probes, which lie outside the deletion. The methylation specific probe for the *NDN* locus, which is affected by the deletion, showed the presence of the methylated maternal allele in the patient, indicating that he has the deletion on his unmethylated paternal allele. In contrast, the father showed no methylation at the *NDN* locus, indicating that he has the deletion on his methylated maternal allele. Since the paternal grandmother has a normal gene dosage in the MLPA analysis, the deletion in the father must have been occurred *de novo*. P, patient; F, father; GM, grandmother.
